# Supplementary material for: Vascular Lipidomic Profiling of Potential Endogenous Fatty Acid PPAR Ligands Reveals the Coronary Artery as Major Producer of CYP450-Derived Epoxy Fatty Acids
Source: Cells. 2020 Apr 29;9(5):1096. doi: 10.3390/cells9051096 (PMC7290345; doi:10.3390/cells9051096)
Supplement: Supplementary file 1 [file cells-09-01096-s001.pdf]

Supplementary Materials:

Table S1. Primer pairs.

pVCAM-1	pCD40L
CCATGGTGTCCCAGAACTTT	GTTTGCCGTCCTGTTGGTAT
TAACTGGGTCCTTGGGTGAG	CTCTCTTTGCCATCCTCCTG
pICAM-1	CYP2J34
TGACCTCCAACATGGAAACA	TTFCTGGAACTGAGACAACG
TCATCAGGAGCTGGGGATAG	GGATGATGTTGCCCATTCTC
pTNFα	β-actin
TCCTCACTCACACCATCAGC	GACATCCGCAAGGACCTCTA
TAGTCGGGCAGGTTGATCTC	ACATCTGCTGGAAGGTGGAC
pMCP-1	
CCGAAGCTTGAATCCTCATC	
TGCTGCTGGTGACTCTTCTG	

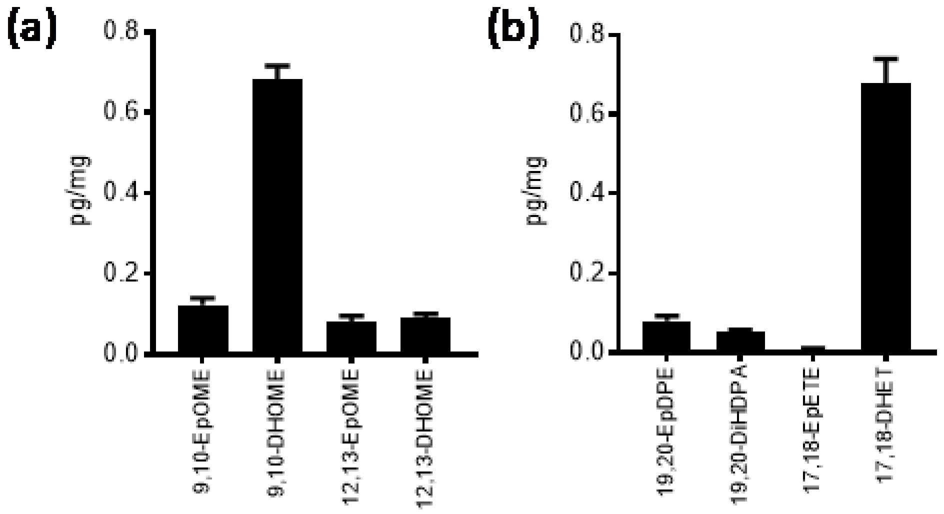

Figure S1. Aorta produces low but detectable levels of CYP-derived oxylipins. Figure shows detectable CYP epoxygenase LA (**a**) and DHA / EPA (**b**) products released by pig aorta. Oxylipins accumulated in 24 h serum free organ culture were measured by LC/MS/MS and expressed as pg/mg of wet tissue weight. Data represents organ culture from n = 3 separate animals.

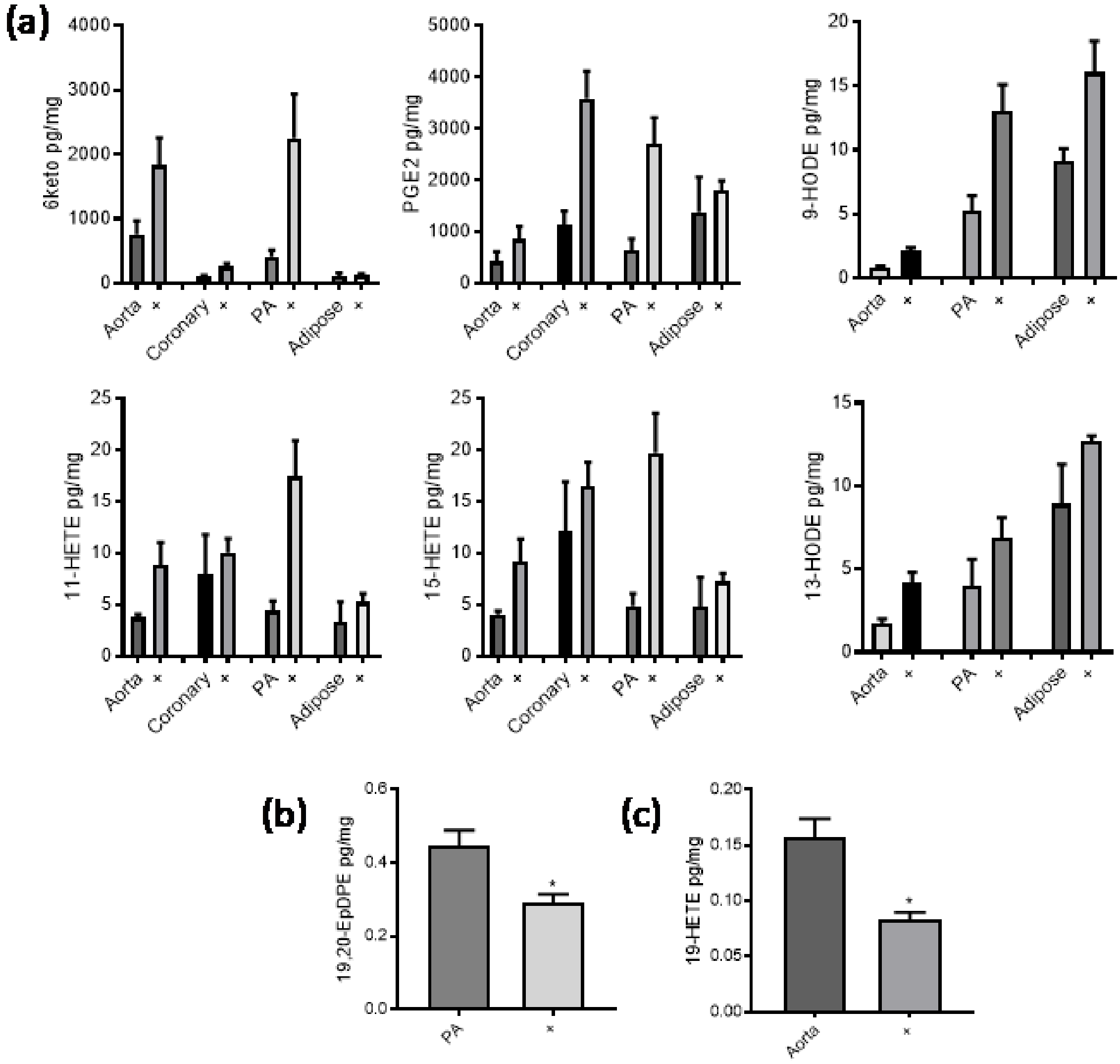

Figure S2. Regulation of oxylipin production in large vessels by LPS/TLR4 activation. (**a**) Comparison of major oxylipin production: 6-keto$PGF_{1\alpha}$, PGE_2, 11-HETE, 15-HETE, 9-HODE, 13-HODE, in aorta, coronary artery, pulmonary artery (PA) and aortic perivascular adipose treated in the absence (-) or presence regulation by LPS (1µg/ml; +). (**b**) 19,20-EpDPE production in pulmonary artery and (**c**) 19-HETE production in aorta, in the absence (-) or presence regulation by LPS (1µg/ml; +). Oxylipins are expressed as mean±s.e.m released in pg/mg tissue produced over 24 h. * indicates $p < 0.05$ by unpaired t-test between tissue treated in the presence of absence of LPS. Data is organ culture from n=3 separate animals.
